# Innate Lymphoid Cells in Mucosal Immunity

**DOI:** 10.3389/fimmu.2019.00861

**Published:** 2019-05-07

**Authors:** Santosh K. Panda, Marco Colonna

**Affiliations:** Department of Pathology and Immunology, Washington University School of Medicine, St. Louis, MO, United States

**Keywords:** ILCs, NK cells, mucosal infections, COPD, IBD – Inflammatory bowel diseases, allergy and asthma

## Abstract

Innate lymphoid cells (ILCs) are innate counterparts of T cells that contribute to immune responses by secreting effector cytokines and regulating the functions of other innate and adaptive immune cells. ILCs carry out some unique functions but share some tasks with T cells. ILCs are present in lymphoid and non-lymphoid organs and are particularly abundant at the mucosal barriers, where they are exposed to allergens, commensal microbes, and pathogens. The impact of ILCs in mucosal immune responses has been extensively investigated in the gastrointestinal and respiratory tracts, as well as in the oral cavity. Here we review the state-of-the-art knowledge of ILC functions in infections, allergy and autoimmune disorders of the mucosal barriers.

## Introduction

ILCs are a family of lymphocytes comprising the innate counterparts of T cells. They are poised to secrete cytokines that respond swiftly to pathogenic tissue damage and shape subsequent adaptive immunity (1). While lacking antigen-specific receptors, ILCs detect changes in the microenvironment through receptors for cytokines that are released upon tissue damage, as well as a broad range of receptors for nutrient components, microbial products, lipid mediators, and neuronal transmitters. Found in both lymphoid and non-lymphoid tissues, ILCs are primarily tissue resident cells and are particularly abundant at the mucosal surfaces of the intestine and lung, whereas they are extremely rare in peripheral blood (2, 3). Based on the signature cytokines they produce, their phenotype, and their developmental pathways, ILCs are divided into three major groups: ILC1s, ILC2s, and ILC3s. Two additional immune cell types, NK cells and lymphoid tissue inducer (LTi) cells, are generally included in the ILC family because their phenotypic, developmental and functional properties overlap considerably with those of ILC1s and ILC3s, respectively (4).

ILC1s secrete IFN-γ in response to IL-12, IL-15, and IL-18. IFN-γ promotes the ability of macrophages and DCs to eliminate intracellular bacteria and to present antigens by inducing expression of MHC and adhesion molecules. The features and functions typical of ILC1s largely overlap with those of NK cells, which also produce IFN-γ. In mice, ILC1s and NK cells both express NKp46 and NK1.1: in humans, both cell types express CD56 and NKp46 (5). A subset of human ILC1s that lacks CD56 and expresses CD127 has also been reported (6). In both humans and mice, ILC1s express markers of tissue residency, such as CD49a and CD103, as well as tissue retention, like CD69, whereas NK cells express markers indicative of recirculation through blood, including CD62L, CCR7, and S1PR (4, 7). Based on higher expression of perforin and granzymes, NK cells have more cytolytic potential than do ILC1s (4, 8). ILC1s can kill target cells through the cell death-inducing molecule TRAIL. In mice, the differentiation of ILC1s requires the transcription factors Hobit and T-bet, whereas NK cells rely on T-bet and Eomes (9–11). However, the expression of these transcription factors in human ILC1s and NK cells does not follow the clear pattern seen in mice. In fact, while human intraepithelial ILC1s express Eomes (5, 12), human hepatic NK cells do not (13), Moreover, differential expression of Eomes during distinct developmental stages of NK cells has been observed (14).

ILC2s, the innate counterparts of Th2, secrete type-2 cytokines such as IL-5, IL-9, IL-13, and amphiregulin in response to TSLP, IL-25, and IL-33. IL-13 plays crucial roles in the expulsion of helminths. Moreover, IL-13 and amphiregulin help repair the tissue damage engendered by helminth and viral infections (15–17). In humans, ILC2s express CRTH2, KLRG1, ST2, and CD25 (18, 19). Although human and mouse ILC2s share many surface markers, expression of CD44 and CD161 differs between the two. Mouse ILC2s express CD44 but not CD161 whereas human ILC2s invert this phenotype and express CD161 but not CD44 (4, 20). ILC2 development depends on the transcription factors GATA3, RORα, and TCF-1 in mice (21–23). GATA3 is also required for maintenance of ILC2 number *in vivo* and its effector function in mice (22, 24). Further, it was demonstrated that GATA3 is required for activation of human ILC2s (25). Although ILC2s are considered homogenous, they are classified into two groups based on their responsiveness to IL-33 and IL-25. IL-33 responsive ILC2s present in the steady state are called natural ILC2s (nILC2), whereas IL-25 responsive ILC2s elicited upon exposure to IL-25 or helminth infection are referred to as inflammatory ILC2s (iILC2) (26, 27). nILC2s are demarcated by elevated expression of Thy1 and ST2 along with relatively little KLRG1; in comparison, iILC2s express more KLRG1, less Thy1 and almost no ST2 on the cell surface (26, 27). Another subpopulation of ILC2s that produce IL-10 (ILC2_10_) has also been identified (28). ILC2_10_ can be induced by IL-33 or papain and are transcriptionally distinct; moreover this population undergoes contraction when the stimulus is removed and the few remaining ILC2_10_ can be promptly activated upon restimulation (28).

**Figure 1:**
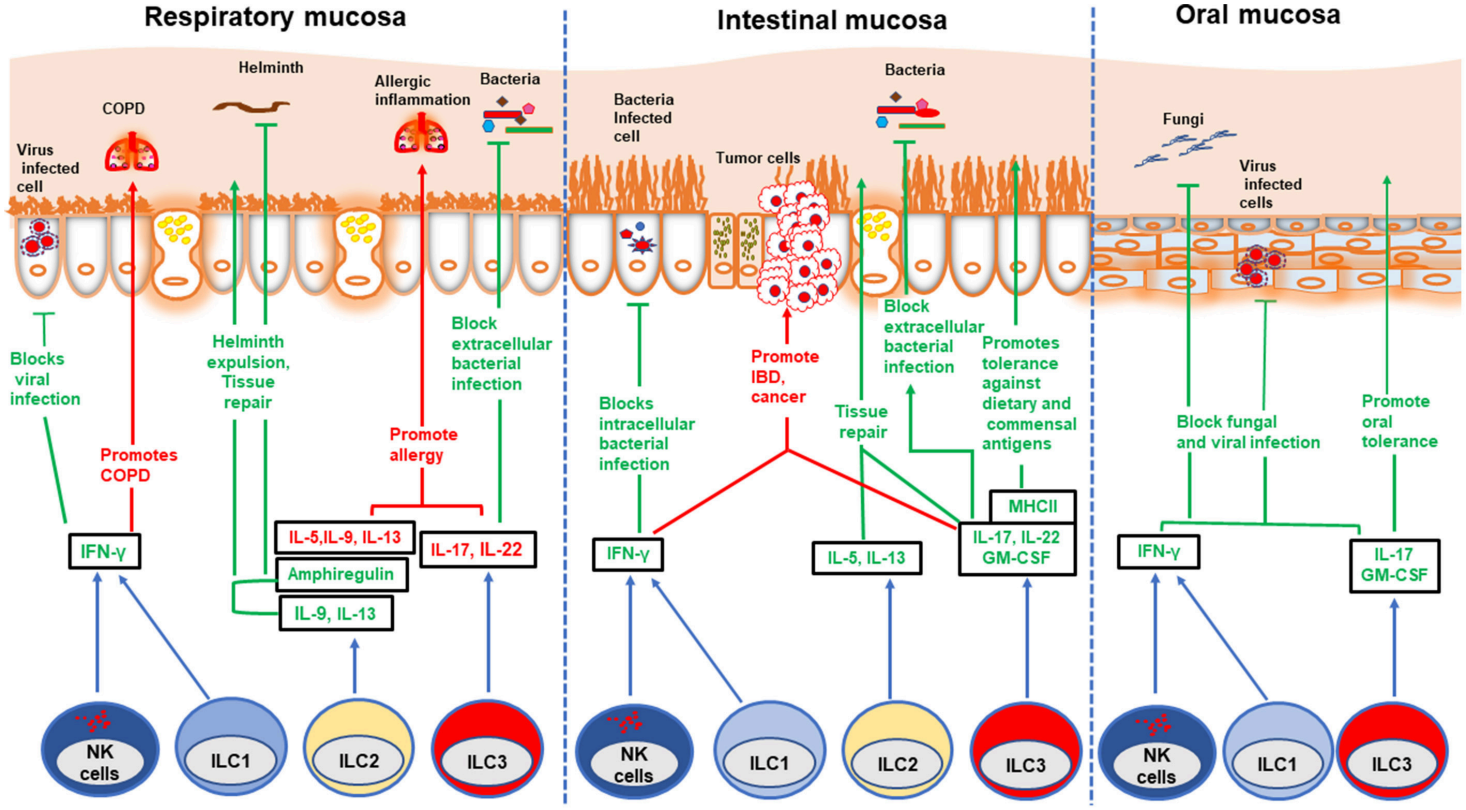
Role of ILCs in different mucosae. Different ILC subsets play beneficial and detrimental roles in different mucosae: protection against various infections, tolerance against innocuous antigens, induction of allergy, IBD, and cancer. NK cells and ILC1s block viral and intracellular bacterial infections by secreting IFN-γ. However, ILC1s promote COPD and IBD in respiratory and intestinal mucosa, respectively. ILC2s contribute to worm expulsion and tissue repair in both respiratory and intestinal mucosa during helminth infection. Further, these cells repair viral induced lung injury by secreting amphiregulin. On the other hand, ILC2s promote allergic inflammation in lung and nasal polyps. ILC3s play dual role in both respiratory and intestinal mucosa. These cells confer protection against extracellular bacterial infection by secreting IL-22 which induces production of anti-microbial peptides by epithelial cells. ILC3s block oral fungal infection in an IL-17-dependent manner. These cells also promote tolerance against commensal and dietary antigens through MHC-II mediated antigen presentation. However, inappropriate activation of ILC3s can play a detrimental role by instigating allergy in the respiratory mucosa, as well as IBD and cancer in the intestinal mucosa.

Because ILC3s produce IL-22 and IL-17 in response to IL-23 and IL-1β, they are considered the innate counterparts of Th17. IL-22 stimulates the secretion of antimicrobial peptides by epithelial cells and mucus production by goblet cells, thereby supporting barrier integrity in the intestine (Figure 1) (29–31). Moreover, it promotes the differentiation of epithelial cells from intestinal stem cells (32). IL-17 promotes granulopoiesis and the secretion of neutrophil chemoattractant (33). ILC3s also produce additional cytokines such as IL-26 (in humans), GM-CSF and TNF-α (34, 35). GM-CSF sustains the generation and survival of myeloid cells, like gut DCs, that promote generation of tolerogenic commensal-specific T cells (35). The differentiation of ILC3s is driven by the transcription factors RORγt and AHR (36, 37). Like ILC3s, LTi are dependent on RORγt and secrete IL-22 and IL-17. In the embryo, LTi drive the development of secondary lymphoid organs, including lymph nodes and Peyer's patches, through the expression of lymphotoxin (LT) that engages LTβ receptor on stromal organizer cells. In the adult, LTi cells cluster to form intestinal cryptopatches, which expand into B cell-rich isolated lymphoid follicles (37–40). In humans, ILC3s express NKp44, CD127, c-Kit, and CCR6. In mice, ILC3s express also CD127, c-Kit, but include CCR6^+^NKp46^−^ LTi as well as CCR6^−^NKp46^−^ and CCR6^−^NKp46^+^ subsets.

Since ILCs mainly populate mucosal sites, this review focuses on how these cells typically contribute to maintenance of barrier integrity and protection against various pathogenic challenges, as well as their propensity to promote allergic and autoimmune diseases when inappropriately stimulated. We individually review the impact of ILCs in the respiratory and gastrointestinal tracts as well as the oral mucosa and discuss potential targeting of ILCs for therapeutic intervention in human diseases.

## ILCs in the Respiratory Tract Mucosa and Lungs

ILCs are present throughout all segments of the respiratory tract. While ILC2s are the predominant ILC population in mice, ILC3s dominate the human respiratory tract (2, 41). However, the composition and functions of ILC subsets dynamically change during experimental mice models of pathology and human diseases.

## ILCs in Respiratory Infections

Experiments in mice have shown that ILC2s promote epithelial and goblet cell proliferation and mucus production in the H1N1 influenza virus infection model through secretion of amphiregulin and IL-13 (15). These ILC2 functions contribute to restore lung epithelial integrity. Accordingly, antibody-mediated depletion of ILC2s compromised lung function during H1N1 influenza infection. However, a study by Chang et al. demonstrated that ILC2s play a pathogenic role through IL-13 production in the H3N1 influenza infection model that induces airway hyper-reactivity, a cardinal feature of asthma (42). H3N1-induced airway hyper-reactivity was attenuated in Il13^−/−^ mice, whereas adoptive transfer of ILC2s restored this response (42). The reason for this discrepancy in the impact of ILC2s is yet to be deciphered; perhaps the differential virulence of the H1N1 and H3N1 viral strains used in these models is responsible. ILC2s also provide defense against helminth infection of the lung through the production of IL-9, IL-13, and amphiregulin (Figure 1) (16, 17, 43). A recent study by Huang et al. demonstrated that the KLRG1^hi^ ST2^−^ ILC2s that protect the lung from helminth-induced tissue damage originate in the intestine and migrate from the intestine to the lung during infection (17).

NK cells and ILC1s contribute to immune responses against viruses through secretion of IFN-γ (Figure 1). NK cells play dual roles during influenza infection. Increased virus accumulation in the lung was observed in the absence of NK cells (44–46). However, depletion of NK cells rendered mice more resistant and able to survive severe influenza infection (47). Furthermore, adoptive transfer of NK cells obtained from influenza-infected lungs into mice newly infected with influenza exacerbated the disease (47). ILC1s protect from Sendai virus and influenza infection by robust secretion of IFN-γ in experimental models (48). Beneficial roles for NK cells, ILC1s and ILC2s have been demonstrated in other mouse models of lung viral infections, such as rhinovirus (49, 50) and respiratory syncytial virus (RSV) (49, 51). Finally, ILC3s have also been implicated in lung infections and inflammation. Lung ILC3s play a protective role in *S. pneumonia* infected mice through secretion of IL-17 and IL-22 (Figure 1) (52). Despite the abundance of studies in mouse models, the role of ILCs in human respiratory infections is yet to be deciphered (Table 1).

**Table 1:** Role of ILCs in different mucosa of humans and mice.

**Mucosa**		**Host**	**Cells**				
			**NK cells**	**ILC1**	**ILC2**	**ILC3**	**References**
**Respiratory**	Infections	Mouse	Protects from viral infections.		Repair the damage induced by viral infections	Blocks extracellular bacterial infections	(45), (48), (15, 52)
		Human	Less number of peripheral NK cells in influenza infected patients	ND	ND	ND	(53)
	COPD	Mouse	NK cells are hyperactivated during COPD	ILC2s converted Into ILC1s	ND	ND	(54, 55)
		Human		Number increased in peripheral blood and lung	ND	Accumulated in the lung tissues of COPD	(40, 41, 54)
	Allergy	Mouse	Inhibits allergic inflammation	Unclear	Promotes allergic inflammation	Induce airway hyperinflammation	(56–58)
		Human	Unclear	Unclear	Increased ILC2 numbers in patients with allergic asthma, rhinosinusitis	Increased ILC3 numbers in patients with allergic asthma	(57, 59, 60)
	Helminth infection	Mouse	Unclear	ND	Worm expulsion and tissue repair	ND	(16, 17)
		Human	Unclear	ND	ND	ND	
**Intestinal**	Infection	Mouse	Blocks intracellular bacterial infection		worm expulsion and tissue repair	Blocks extracellular bacterial infection	(39, 61–64)
		Human	ND	ND	ND	ND	
	Homeostasis	Mouse	ND	ND	ND	Maintain tissue homeostasis	(32, 35)
		Human	ND	ND	ND	ND	
	IBD	Mouse	Unclear	Promotes IBD	ND	Promotes IBD	(5, 65, 66)
		Human	Unclear	Accumulated in the inflamed gut	ND	Accumulated in the inflamed gut	(5, 67)
	GVHD	Mouse	Promotes/inhibits	ND	Promotes barrier integrity	Promotes barrier integrity	(68–70)
		Human	Unclear	ND	ND	Reverse correlation between the number of ILC3 and disease severity.	(70, 71)
**Oral**	Infection	Mouse	ND	ND	ND	Blocks fungal Infection and promote tolerance against dietary antigens	(35, 72)
		Human	ND	ND	ND	ND	

*ND, Not defined*.

## ILCs in Asthma

Multiple studies concur that ILC2s play a pathogenic role during lung allergy and inflammation (Figure 1) (73, 74). Mice lacking ILCs, T cells, and B cells developed less airway inflammation than did mice lacking only T cells and B cells in the papain-induced eosinophilic asthma model (56, 75). Furthermore, adoptive transfer of ILC2s was sufficient to induce airway inflammation in the absence of B and T cells (75). ILC2s initiate allergic lung inflammation by secreting IL-13, which promotes migration of activated DCs to the draining lymph node where they prime Th2 differentiation (56). Lung ILC2s promoted proliferation and antibody production by B1 and B2 cells *in vitro* and contributed to enhanced production of IgM *in vivo* in an experimental model of polysaccharide-mediated nasal allergy (76). Moreover, ILC2s acquired a memory-like phenotype after primary encounter with allergen and more readily promoted allergic inflammation in the lung upon secondary encounter with unrelated allergens than did naïve ILC2s (77).

In human, expansion of ILC2s and elevated type-2 cytokines have been observed in a variety of patients with allergic asthma (Table 1). Approximately twice as many ILC2s were found in the peripheral blood of allergic asthmatic patients than in healthy controls (60). In addition, ILC2s from allergic asthmatic patients were hyper-responsive to IL-33 and IL-25 and secreted significantly higher amounts of IL-5 and IL-13 than did ILC2s from healthy controls (60). Smith et al. reported higher percentages of ILC2s in the blood and sputum of prednisone-dependent severe eosinophilic asthma patients than in the blood of asymptomatic patients with steroid-naive mild atopic asthma or healthy controls (78). Respiratory allergen challenge of patients with allergic asthma elicited a sudden increase and rapid activation of ILC2s primed to secrete IL-5 and IL-13 (79). Further supporting a role for ILC2s in allergic asthma, the frequency of ILC2s and the levels of IL-33 in the bronchoalveolar lavage (BAL) of patients with allergic asthma were positively correlated with severity of the disease (80). Likewise, the percentage of ILC2s was higher in the peripheral blood of patients with eosinophilic asthma and positively correlated with eosinophil counts in the sputum (81). Similarly, the frequency of ILC2s was higher in the blood and BAL of children with severe therapy-resistant asthma than in children with recurrent lower respiratory tract infections (82). Finally, an increase of ILC2s in the BAL of patients with idiopathic pulmonary fibrosis has also been observed (83).

How other ILCs impact asthma is less clear. ILC3s have been suggested to play a pathogenic role in asthma patients. Numbers of IL-17-producing ILC3s in the human lung positively correlate with severity of the disease (57). IL-22 production by ILC3s has also been implicated in asthma pathogenesis, as asthma patients have increased levels of IL-22 compared to healthy controls (84). In mice, NK cells and ILC1s that produce IFN-γ inhibit ILC2 expansion and type-2 cytokine production (85, 86) and hence might help control the disease. Consistent with this, depletion of NK cells during the early stages of papain-induced lung allergy in mice resulted in increased ILC2-mediated cytokine production, which exacerbated pathology (58). Furthermore, Han et al. demonstrated that IFN-γ inhibits expansion of IL-13 producing ILC2s and attenuates the development of rhinovirus-induced asthma pathology in baby mice (87). Altogether, these studies suggest that ILC1s and NK cells attenuate the initiation and progression of allergic inflammation in the lung in mouse models. However, the role of NK cells in human allergic asthma is unclear. A human study documented fewer NK cells in the peripheral blood of severe asthma patients than in healthy controls, as well as an enrichment of the CD56^bright^ NK cell subset in the BAL of asthmatic patients, whereas no significant difference in the frequency of ILC2s was detected in the blood of controls, severe, and mild asthma patients (88). One report described increased frequency of an unusual population of NK cells that produces IL-4 rather than IFN-γ in the blood of asthmatic patients (89). In contrast, another study showed that heightened IFN-γ responses in the airways of patients were associated with severe asthma, which often involves mixed granulocytic inflammation that includes neutrophils as well as eosinophils (90). Supporting a role of IFN-γ in initiation of airway hyper responsiveness these authors showed that in a mouse model mimicking human severe asthma IFN-γ^−/−^ mice failed to exhibit the symptoms of severe asthma as compared to wild type controls (90). Further research in human patients focusing on NK cells, ILC1s, and ILC2s in both blood and inflamed tissue might decipher how the balance between different subsets of ILCs impacts asthma pathogenesis.

## ILCs in Allergic Rhinitis

ILC2s have also been implicated in the pathogenesis of allergic rhinitis. The frequency of ILC2s was elevated in nasal polyps characteristic of chronic rhino-sinusitis and aspirin-exacerbated respiratory disease and positively correlated with disease severity (18, 41, 59, 91, 92). Recruitment of ILC2s to the nasal mucosa upon a nasal mucosal challenge in subjects with allergic rhinitis was demonstrated in human volunteers (93). TSLP produced by epithelial cells and IL-4 produced by eosinophils induces ILC2 activation and secretion of IL-4, IL-5, and IL-13; this activates eosinophils and thereby elicits a positive feedback loop. Disrupting this loop with anti-IL-5 antibodies was beneficial for treating chronic rhino-sinusitis in humans (94, 95). More ILC2s have been noted in the peripheral blood of allergic rhinitis patients than in blood from healthy controls (96, 97). Moreover, the response to house dust mite-specific immunotherapy negatively correlated with ILC2 numbers; patients with a good response to the allergen-specific immunotherapy had relatively few ILC2s (97). Furthermore, subcutaneous allergen therapy resulted in fewer circulating ILC2s in patients with allergic rhinitis (98). In contrast, NK cells appear to play a beneficial role in allergic rhinitis, as patients had fewer circulating CD56^bright^ NK cells than did healthy controls (99).

## ILCs in Chronic Obstructive Pulmonary Disease of the Lung and Cancer

Chronic obstructive pulmonary disease (COPD) is a disorder caused by cigarette smoke or long-term inhalation of noxious gases, which damage the upper and lower airways, eventually causing bronchitis and emphysema (100). Studies in mice and humans have demonstrated that lung resident ILC2s acquire an ILC1 phenotype and secrete IFN-γ during COPD (41, 54). Furthermore, ILC1 numbers were increased in the peripheral blood of patients with severe COPD (Table 1). Additionally, a high ILC1-to-ILC2 ratio in the blood of COPD patients positively correlated with severity of the disease (54). Shikhagaie et al. reported significantly increased numbers of neuropilin^+^ ILC3s in the lungs of patients with COPD, which were associated with formation of ectopic lymphoid aggregates (40). Further supporting a role for ILC3s in COPD, IL-17 was found to accumulate in the lungs of COPD patients (101).

In contrast to their deleterious effect in COPD, ILCs may be beneficial in lung cancer. ILC3s have been reported to congregate in human non-small cell lung carcinoma tissues where they may play a protective role by promoting the formation of tertiary lymphoid structures (102). The role of NK cells in lung cancer remains confusing, but should become more well-defined as better reagents/methods to detect NK cells and relevant activating and inhibiting receptors are developed and widely used. An early study that employed CD57, which is not specific for NK cells, found that the presence of tumor infiltrating CD57^+^ cells was associated with a better prognosis in patients with primary squamous cell lung carcinoma (SCLC) (103). A more recent study suggested that infiltration of CD16^+^CD56^+^ NK cells in the tumor periphery correlated with increased survival time of patients with non-small-cell lung carcinoma (NSCLC) (104). This study also noted that intra-tumoral NK cells downregulated activating receptors, including NKG2D, and upregulated the inhibitory receptor NKG2A in a mouse model of metastatic human large cell lung cancer. Consistent with this, Platonova et al. reported that intra-tumoral NK cells in NSCLC patients have an altered phenotype, with decreased expression of activating receptors such as NKp30, NKG2D, NKp80 and increased expression of the inhibitory receptor NKG2A (105). Along these lines, Carrega et al. found that most NK cells infiltrating NSCLC were CD56^bright^ CD16^−^; the intra-tumoral NK cells had less cytotoxic potential than did NK cells in the peripheral blood and lung, but retained the ability to secrete IFN-γ and TNF-α (106). However, Hodge et al. reported that NK cells (as well as NKT, CD4^+^ and CD8^+^ T cells) isolated from lung cancer tissue secreted less perforin, granzyme B, IFN-γ, TNF-α after stimulation than did NK, NKT, and T cells isolated from healthy tissue (107). Finally, Christmas et al. found that while the frequency of peripheral NK cells did not differ between patients with NSCLC or SCLC and healthy controls, NK cells isolated from cancer patients had diminished expression of NKp46, CD25, and perforin A and were functionally impaired in terms of cytoxicity and cytokine production (108). Collectively, these studies suggest that the tumor microenvironment either recruits NK cells with limited cytotoxic potential or, more likely, directly alters the phenotype of NK cells in order to escape from NK cell-mediated anti-tumor responses. Further research in human patients is warranted to delineate the precise role of ILCs in lung cancer.

## The Impact of ILCs on Intestinal Mucosa

ILC3s are the most abundant subset of ILCs in both the fetal and adult human intestine (8). In contrast, the distribution pattern of ILC1s and ILC2s changes during development. In the fetal intestine ILC2s prevail over ILC1s, whereas ILC1s assume the dominant role in the adult intestine (8, 18). ILCs are unevenly distributed throughout the segments of the gastro-intestinal (GI) tract. The upper GI tract, e.g., esophagus, is enriched with ILC1s, whereas the distal part of the ileum and colon are chiefly populated by ILC3s (109).

## ILCs in Gastrointestinal Tract Infections

The role of ILCs in intestinal infection has been demonstrated in various experimental models (Table 1); ILC3s participate in the immune responses to *Citrobacter rodentium, Clostridium difficile, Salmonella enterica, Listeria monocytogenes, and Toxoplasma gondii* (10, 39, 63, 110–112). Further work has shown that ILC3s restrict colonization of segmented filamentous bacteria in the gut (113) and inhibit the invasion of *Alcaligenes* species into Peyer's patches (114). ILC3s counter bacterial infections through secretion of IL-22, which stimulates epithelial cells to produce antimicrobial peptides (Figure 1) (31). Furthermore, IL-22 induces fucosyltransferase 2 expression in intestinal epithelial cells, which promotes fucosylation of surface proteins that are shed into the lumen. These fucosylated proteins release fucose, which is metabolized by microbes as an energy source. Fucose metabolism has been shown to suppress virulent gene expression in commensal bacteria (115), and Goto et al. demonstrated that the absence of epithelial cell fucosylation renders mice more susceptible to *Salmonella* infection (62). IL-22 production is mainly stimulated by IL-23 and IL-1β produced by inflammatory monocytes, macrophages and DCs. TNF-like ligand 1A produced by mononuclear phagocytes upon interaction with microbiota in the gut also induces IL-22 production by ILC3s and promotes healing of mucosal layer during DSS-induced acute colitis (116).

ILC1s and NK cells help combat intracellular pathogens such as *T. gondii, L. monocytogenes, Salmonella typhimurium* and viruses through IFN-γ production (10, 117, 118). ILC1s cooperate with ILC3s in providing protection against intestinal *C. difficile* infection (112); accordingly, rapid infection and death ensue in the absence of IFN-γ and IL-22. By secreting IL-5 and IL-13, ILC2s induce goblet cell differentiation and mucus production that propels worm expulsion and protects from tissue damage (61, 64, 119, 120). Finally, while most studies have focused on bacterial infections, ILCs may also impact intestinal viral infections. Studies in mice have shown a protective role for ILC3s during rotavirus infections, in which IL-22 synergizes with IFN-λ in inducing STAT1 activation (121). Recently, a cross-sectional study reported that the colons of HIV-1 infected patients are enriched with NKp44^+^ILC1s that produce IFN-γ (122).

While the above studies support a critical role for ILCs in maintaining barrier integrity that protects mice from a variety of infections, one study has suggested that ILCs are completely redundant. Vely et al. studied SCID patients following allogeneic hematopoietic stem cell transplants that reconstituted the T cell compartment but not the ILC compartment. These patients did not manifest an increased risk of infections and other complications (123). Further research in human subjects addressing these issues is warranted.

## ILCs in Autoimmunity

ILCs have been extensively studied in intestinal autoimmune disorders, particularly inflammatory bowel disease (IBD) (Table 1). Crohn's disease (CD) and ulcerative colitis (UC) are the two main forms of IBD that affect distinct layers of the gut wall and colonic mucosa. Although the mechanisms underpinning IBD remain incompletely understood, immune responses against commensal microbes in the intestine have long been implicated. The role of ILC3s in IBD is unclear. Several papers have proposed that ILC3s may contribute to autoimmunity. IL-23, the key activator of ILC3s, has been shown to drive colitis (124). Accumulation of ILC3s in inflamed colons of *Helicobacter hepaticus* infected mice has been reported (65). Moreover, mice either depleted of or genetically modified to lack ILC3s were resistant to colitis (65). Similarly, IL-17-producing ILC3s have been shown to drive colitis, whereas administration of anti-IL-17 antibodies ameliorated disease (125). ILC3s also drove intestinal inflammation and pathology in the anti-CD40 model of innate colitis mice through secretion of IL-22 and GM-CSF (66). In agreement with this study, Pearson et al. demonstrated that ILC3-mediated production of GM-CSF recruits inflammatory monocytes into the intestine, which sustain intestinal inflammation (126). Corroborating these findings in humans, IL-17-producing ILC3s have been found to accumulate in the inflamed colons of patients with CD (127). Other studies have proposed a tolerogenic role for ILC3s during intestinal inflammation. ILC3s express MHC class II and can present commensal bacteria to T cells. However, because they lack costimulatory molecules, ILC3s are unable to elicit commensal-specific CD4^+^ T cells and, in fact, induce T cell tolerance and mitigate inflammation in mouse models (128, 129). Consistent with this, unusually low expression of MHC-II has been observed on colonic ILC3s from pediatric IBD patients (128). A similar role for ILC3s in maintaining tolerance against dietary and innocuous commensal antigens has also been reported (35, 129, 130).

ILC1s have also been implicated in human IBD (Table 1). CD127^+^ lamina propria ILC1s and CD127^−^ intra-epithelial ILC1s amass in the inflamed intestine of CD patients (5, 8). Interestingly, expansion of IFN-γ producing cells with an NK cell phenotype in the lamina propria of CD patients was accompanied by a reciprocal decrease in IL-22 producing NKp44^+^ ILC3s (131). Li et al. also observed that the frequency of ILC1s rises in the inflamed ileum of CD patients, whereas frequency of ILC3s declines (132). The increase in ILC1 frequency at the cost of ILC3s might be due to plasticity of ILC3s, which transdifferentiate into ILC1s under the influence of IL-12 and/or IL-23 produced by myeloid cells (12). Increased frequency of Lin^−^CD127^+^ ILC1s in the colons of patients with IBD and primary sclerosing cholangitis has also been observed. Furthermore, these authors have demonstrated that the frequency of ILC1s drops in the blood of patients, suggesting that ILC1s are recruited into the inflamed tissue (133).

ILC2s have also been implicated in intestinal inflammation. Expansion of IL-13 producing ILC2s has been shown to play a detrimental role in oxazolone-induced colitis (134). However, expansion of amphiregulin-producing ILC2s has been shown to contribute to tissue repair during DSS-induced colitis (135). Thus, ILC2s may either promote or block intestinal pathogenesis, depending on the disease setting. ILC2s have been noted in intestinal samples from CD patients (19). Interestingly, these ILCs secreted IFN-γ, reflecting the plasticity of ILC2s during intestinal inflammation. Increased ILC2 frequency that positively correlates with severity of the disease has been observed in patients with eosinophilic oesophagitis (136). While accumulation of ILCs in the intestine of CD patients has been demonstrated, whether ILCs are involved in UC remains unclear. In fact, Gwela et al. reported no difference in ILC frequencies between UC patients and healthy controls (133).

In addition to autoimmunity, intestinal tissue damage can be induced by chemotherapy and radiotherapy. In this case, ILC3s help repair intestinal damage by secreting IL-22 (68, 137). During graft-vs.-host disease (GVHD), which can occur in leukemic patients after allogeneic hematopoietic stem cell transplant, allogeneic T cells can attack and destroy ILC3s, thereby depleting intestinal IL-22. In mice with acute GVHD, ILC3s secreted significantly less IL-22, resulting in impaired epithelial functions (Table 1) (68). Corroborating a role for ILC3s and ILC3-secreted IL-22 in tissue repair and resolution of inflammation during GVHD, fewer ILC3s were observed in patients with acute or chronic GVHD than in patients that did not develop GVHD, (71). Furthermore, recovery of gut homing CD69^+^ activated ILC3s was associated with a lower incidence of GVHD (71). Finally, infusion of donor ILC2s after bone marrow transplant reduced the lethality of GVHD up to 70% and enhanced the barrier function of the GI tract in an allogenic stem cell transplant model (69). Altogether, these studies support a beneficial role for ILC2s and ILC3s in prognosis of GVHD.

## ILCs in Intestinal Cancer

It has been known for long time that IBD patients have an increased risk of intestinal cancer due to chronic inflammation (138). Given that ILCs accumulate in the intestinal mucosa during IBD, they could contribute to the inflammatory environment and may play a pro-tumorigenic role. Indeed, the key activator of ILC3s, IL-23, and the ILC3 effector cytokines IL-17 and IL-22 are associated with both experimental and clinical tumorigenesis (Figure 1) (139–142). In contrast to ILC3s, NK cells and ILC1s may have anti-tumorigenic effects through secretion of IFN-γ. A decreased frequency of NK cells expressing NKp30, NKp46, and NKG2D paralleled by an increased frequency of ILC2s was noted in the peripheral blood of gastric cancer patients in comparison to healthy controls (143). Similarly, serum levels of IFN-γ in gastric cancer patients were lower than those found in healthy donors (144, 145). Although infiltration of NK cells into gastric cancer tissues is scarce (146), the presence of intratumoral NK cells correlated with a better prognosis (147). While these studies suggest that ILCs are involved in the pathogenesis of intestinal cancer, further studies in human patients is required to address their exact role.

## ILCs in the Oral Mucosa

Like the intestinal mucosa, the oral mucosa is populated with commensal microflora and exposed to dietary antigens and pathogens. Thus, ILCs present in the oral mucosa may help maintain barrier function and protect against pathogenic infections. Human ILC3s and intraepithelial ILC1s were originally identified in tonsils, which are secondary lymphoid tissues coated by the oral mucosa (5, 34). ILCs have been also found in human gingivae. Approximately 10–15% of total CD45^+^ cells identified were ILCs and most of them were IFN-γ secreting ILC1s (148). A recent study by Brown et al. demonstrated the presence of all three groups of ILCs in the murine gingivae (149). ILC3s provided protection against oropharyngeal infection with *Candida albicans* through production of IL-17A and IL-17F induced by IL-23 (Figure 1) (72); thus, both *Ror*γ*t*^−/−^ mice lacking ILC3s and mice depleted of ILC3s by antibody treatment suffered severe infection (72). IFN-γ-producing ILC1s and NK cells have been found in the oral mucosa of macaques. During simian immunodeficiency virus infection, expansion of ILC1s and increased IFN-γ production were noted in the oral draining lymph nodes and tonsils whereas NK cells remained unchanged (150). Future research will decipher the impact of ILCs in oral immune responses to commensals and dietary antigens.

## Targeting ILCs for Therapy

**Figure 2:**
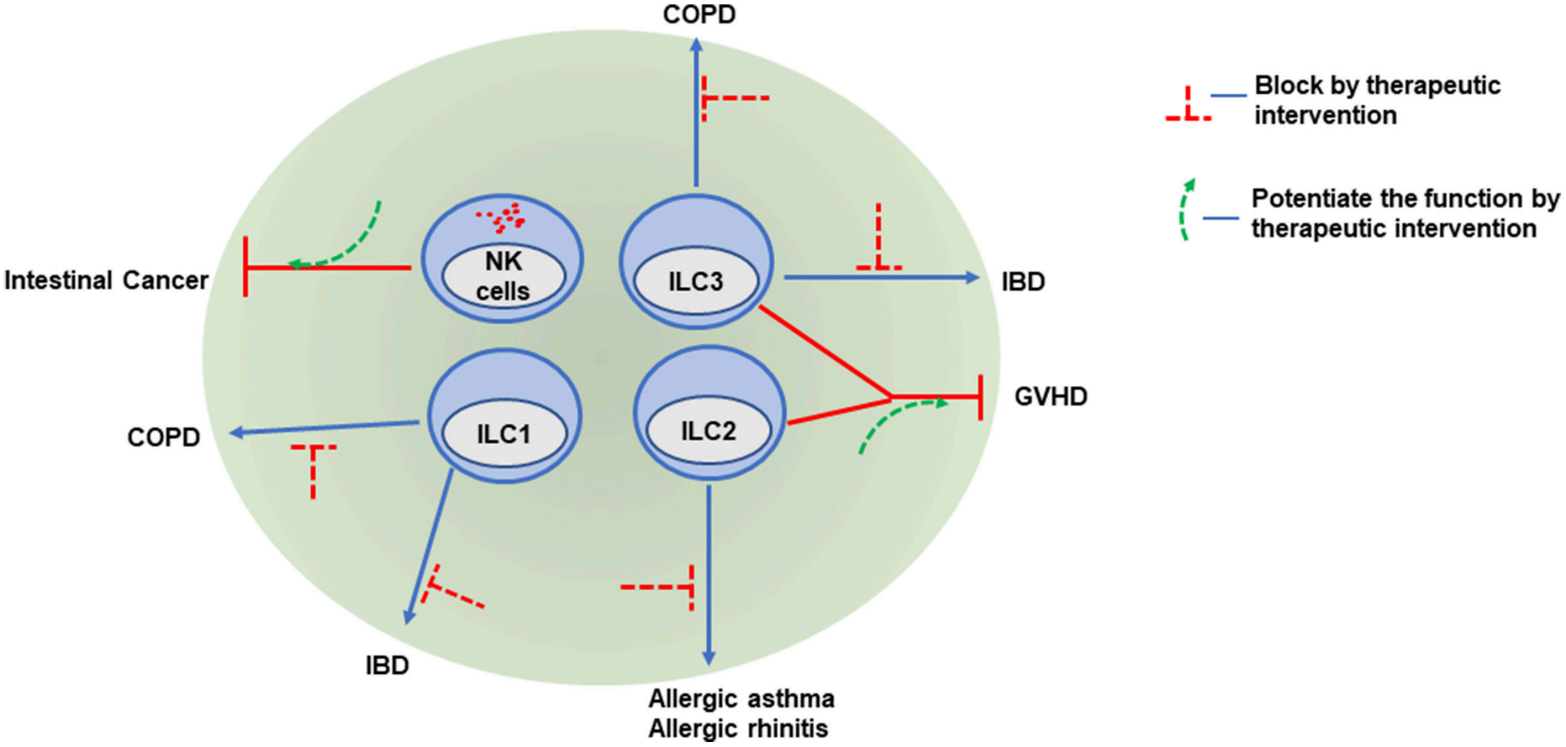
ILCs as potential therapeutic targets. Because of their various roles in promoting or attenuating pathogenesis, ILCs may be potential therapeutic target. Infiltration of NK cells into intestinal cancer is associated with better prognosis, therefore increasing NK cell infiltration and potentiating NK cell functions in tumors might be beneficial. Hyperactivation of both ILC1 and ILC3 promote COPD and IBD. Regulation of pathogenic cytokine production or antibody mediated depletion of these cells might be a potential treatment option. Similarly, antibodies targeting type-2 cytokines produced by ILC2 may be promising in the treatment of allergy and asthma patients. Both ILC2 and ILC3 ameliorate clinical symptoms and play protective roles in GVHD. Expansion of ILC2 and ILC3 numbers by therapeutic intervention might be helpful to control GVHD.

A strong rationale for developing therapies specifically targeting ILCs, such as depleting or agonistic antibodies, depends on whether ILCs play unique, non-redundant roles in the context of various diseases, which is yet to be firmly established. However, evidence from both experimental mice models and humans has suggested that therapeutic targeting of ILCs might be beneficial for autoimmune disorders. Depletion of Thy-1 positive ILCs was beneficial for treating *H. hepaticus* induced colitis (65). Similarly, depletion of ILC2s blocked the development of papain-induced allergy (75). When ILCs and T cells have redundant functions, an alternative option is to neutralize the effector cytokines produced by both ILCs and T cells. Blockade of the IL-17 receptor or neutralizing IL-17 and IFN-γ in several clinical trials failed to ameliorate IBD (151, 152). However, targeting both IL-17 and IFN-γ bore promising results in preclinical models (65). Blockade of cytokines that stimulate IL-17 and IFN-γ secretion, such as IL-12 and IL-23 or IL-23 alone, has also been effective for treating CD patients (153, 154). Since GVHD is associated with fewer IL-22-producing ILC3s, enhancement of ILC3 numbers and function might be explored for the treatment of GVHD (Figure 2) (68, 71).

Several attempts have been made to explore the efficacy of targeting ILC2s in respiratory diseases. Anti-IL-5 and anti-IL-4 receptor α treatments have had promising effects in treating patients suffering from chronic rhinosinusitis with nasal polyps and eosinophilic asthma (94, 155, 156). Similarly, inhibiting ILC2 functions with CRTH2 antagonists can restore lung function in asthma patients (Figure 2) (157, 158). Further research and well-designed clinical studies should help delineate the efficacy of targeting ILCs in various mucosal diseases.

## Future Perspectives

ILCs appear to contribute significantly to human health and disease, playing beneficial roles in some mucosal infections and GVHD, while aggravating pathology in IBD and COPD. Are ILCs a valid therapeutic target? To address this question, it is important to understand whether ILCs play unique roles or are largely redundant with T cells. Studies in mouse models have provided limited answers, as they are mainly performed in RAG-deficient mice that lack adaptive responses. Animal models in which ILCs are selectively ablated in the presence of intact adaptive responses are underdeveloped. Most human studies describe single timepoint assays that provide a snapshot of ILCs at a particular stage of the disease. Longitudinal studies addressing multiple subsets of ILCs at different stages of disease is warranted to provide a clearer picture of their impact. For instance, while NK cells seem to control lung ILC2 expansion in asthma, it is important to understand whether NK-ILC2 cross-talk occurs at a particular stage or throughout the disease. Similarly, ILC3 secretion of IL-17 and IFN-γ seems to be detrimental in IBD, whereas IL-22 secretion may be protective. Thus, it is essential to examine the ratio of ILC3s producing IL-17/IFN-γ and IL-22 at different stages of the disease. Although ethical constraints and limitations of available tissue restrict human studies, further analysis including diverse age groups and focusing on different stages of disease would help better understand the role of ILCs as well as how they might be targeted for the therapy of mucosal diseases.

## Author Contributions

SP wrote the first draft. MC contributed to conceiving and writing the manuscript.

### Conflict of Interest Statement

MC receives research support from Pfizer. The remaining author declares that the research was conducted in the absence of any commercial or financial relationships that could be construed as a potential conflict of interest.
